# RNAi Knockdown of *EHMT2* in Maternal Expression of Prader–Willi Syndrome Genes

**DOI:** 10.3390/genes15111366

**Published:** 2024-10-24

**Authors:** Violeta Zaric, Hye Ri Kang, Volodymyr Rybalchenko, Jeffrey M. Zigman, Steven J. Gray, Ryan K. Butler

**Affiliations:** 1 Department of Psychiatry, UT Southwestern Medical Center, Dallas, TX 75390, USA; violeta.zaric@utsouthwestern.edu (V.Z.); volodymyr.rybalchenko@utsouthwestern.edu (V.R.); jeffrey.zigman@utsouthwestern.edu (J.M.Z.); 2 Department of Pediatrics, UT Southwestern Medical Center, Dallas, TX 75390, USA; hyeri.kang@utsouthwestern.edu (H.R.K.); steven.gray@utsouthwestern.edu (S.J.G.); 3 O’Donnell Brain Institute, UT Southwestern Medical Center, Dallas, TX 75390, USA; 4 Center for Hypothalamic Research, Department of Internal Medicine, UT Southwestern Medical Center, Dallas, TX 75390, USA

**Keywords:** Prader–Willi Syndrome, RNAi, EHMT2, SNORD116, SNRPN

## Abstract

Background/objectives: Euchromatic histone lysine methyltransferase 2 (EHMT2, also known as G9a) is a mammalian histone methyltransferase that catalyzes the dimethylation of histone 3 lysine 9 (H3K9). On human chromosome 15, the parental-specific expression of Prader–Willi Syndrome (PWS)-related genes, such as *SNRPN* and *SNORD116*, are regulated through the genetic imprinting of the PWS imprinting center (PWS-IC). On the paternal allele, PWS genes are expressed whereas the epigenetic maternal silencing of PWS genes is controlled by the EHMT2-mediated methylation of H3K9 in PWS-IC. Here, we measured the effects of RNA interference of EHMT2 on the maternal expression of genes deficient in PWS in mouse model and patient iPSC-derived cells. Methods: We used small interfering RNA (siRNA) oligonucleotides and lentiviral short harpin RNA (shRNA) to reduce *Ehtm2*/*EHMT2* expression in mouse *Snord116* deletion primary neurons, PWS patient-derived induced pluripotent stem cell (iPSC) line and PWS iPSC-derived neurons. We then measured the expression of transcript or protein (if relevant) of PWS genes normally silenced on the maternal allele. Results: With an approximate reduction of 90% in *EHMT2* mRNA and more than 80% of the EHMT2 protein, we demonstrated close to a 2-fold increase in the expression of maternal transcripts for *SNRPN* and *SNORD116* in PWS iPSCs treated with si*EHMT2* compared to PWS iPSC siControl. A similar increase in *SNORD116* and *SNRPN* RNA expression was observed in PWS iPSC-derived neurons treated with sh*EHMT2*. Conclusions: RNAi reduction in EHMT2 activates maternally silenced PWS genes. Further studies are needed to determine whether the increase is therapeutically relevant. This study confirms the role of EHMT2 in the epigenetic regulation of PWS genes.

## 1. Introduction

Prader–Willi Syndrome (PWS, OMIM176270) is an imprinted neurodevelopmental disorder. Major manifestations include early childhood obesity, hyperphagia, hypotonia with poor suck and poor weight gain in infancy, mild-to-moderate intellectual disability, hypogonadism, growth hormone insufficiency, and, frequently, psychiatric disturbance, including abnormal restricted repetitive behavior (e.g., skin picking, obsession, compulsion, sameness behavior, etc.). PWS is caused by the loss or loss-of-function of genes normally expressed on the paternal allele located in the chromosomal region 15q11-q13. Paternal expression is regulated by the PWS imprinting center (PWS-IC), whereas genes in this same region are epigenetically repressed due to the imprinting mechanism on the maternal allele. In most cases (70%), the large deletion (LD) of paternal 15q11-q13 region, comprised of several expressed protein-coding genes (*MAGEL2*, *SNURF*, and *SNRPN*, among others), a cluster of C/D box small nucleolar RNA noncoding genes (including *SNORD116* and *SNORD115*), and several long noncoding transcripts (including *IPW* and the antisense transcript to *UBE3A*), leads to PWS pathogenesis [1,2,3]. Approximately 25% cases are due to the uniparental disomy (UPD) of maternal chromosome 15 [4,5], and ~1% of cases are due to aberrant DNA methylation throughout the imprinted domain on chromosome 15 [6]. Patients with the small deletion (SD) of 108 kb encompassing *SNORD116* cluster and *IPW* genes appear to have most of the PWS-related clinical phenotypes, which shows the importance of *SNORD116* in PWS pathogenesis [7,8,9,10]. Other rare PWS cases presenting the PWS symptoms of overeating and obesity have emerged and have been described with small, atypical deletions overlapping PWS-IC or a partial deletion of *SNURF*-*SNRPN*, or even a microdeletion of 78 kb that includes *SNURF*-*SNRPN* exons 2 and 3 [11,12,13]. However, although, in these last cases, patients do not express *SNORD116*, these patients do not manifest all PWS classical symptoms, as encountered in PWS patients with LD. Presently, there is no cure for PWS, and existing treatments address individual symptoms, necessitating a complex and burdensome regimen. There is a need for genetic therapies for PWS that can address the root cause of the disease and comprehensively mitigate the multitude of symptoms through a single treatment.

New methods of epigenetics-based intervention for the treatment of PWS targeting DNA methylation at the PWS-IC [14,15] or histone methylation at the *SNORD116* locus have recently emerged [16]. In general, DNA methylation and histone lysine methylation are described as important epigenetic tools in maintaining gene silencing in numerous cellular processes, including embryonic development, genomic imprinting, X-chromosome inactivation, and the preservation of chromosome stability [17,18]. Particularly, the dimethylation of lysine 9 of histone H3 (H3K9me2) by euchromatic histone lysine N-methyltransferase-2 (EHMT2, also known as G9a) is associated with transcriptional repression and regulating genomic imprinting in PWS [19]. Indeed, not only is the PWS-IC on the maternal chromosome 15 found to be a methylated CG-rich region but H3K9me2 also acts as a repressor of maternal PWS gene expression in the 15q11-q13 chromosomal region. EHMT2 is essential in mouse embryonic development. Deletion and loss-of-function mutations result in lethality and severe growth defects by embryonic day 9.5. However, a neuron-specific deficiency in *Ehmt2* did not reveal obvious neuronal developmental or architectural defects. Furthermore, a postnatal conditional genetic reduction in *Ehtm2* does not affect skeletal muscle development [20] and reduces pain- and anxiety-like behaviors in mice [21,22,23]. Kim et al. recently demonstrated that the small molecules UNC0638 and UNC0642 can selectively reduce H3K9me2 [24]. The authors showed the activation of maternal PWS imprinted genes, including the cluster *SNORD116*, in fibroblasts from PWS patients and in a PWS mouse model without changing the methylation rate at the PWS-IC. This supports the idea that the activation of maternal genes is independent of PWS-IC DNA methylation. We are interested in pursuing epigenetics-based therapy by exploring the feasibility of the long-lasting gene downregulation of *EHMT2* using short hairpin RNA (shRNA) expressed from a vector, modeled in cultured cells using either plasmids or lentivirus. shRNA, expressed as double-stranded RNA stems (19–29 bases) and joined by a short hairpin loop sequence, is processed by Dicer and incorporated into the RNA-induced silencing complex to become a small interfering RNA (siRNA), resulting in the targeting and degradation of cognate mRNAs [25,26,27]. Compared to siRNA, the benefit of shRNA is that it can be incorporated into permanent expression vectors and offer a long-standing effect on cellular function in therapeutic applications while maintaining a high level of specificity in gene silencing. Moreover, shRNA has also been widely used in research for over a decade, and there are currently five RNA interference-based drugs that have been approved by the FDA [28]. Our objective here is to test the hypothesis that a vector-derived anti-*EHMT2* shRNA strategy is effective in elevating the maternal expression of PWS genes in vitro.

## 2. Materials and Methods

### 2.1. Animal Procedures

All animal studies were conducted in accordance with IACUC and under UT Southwestern Medical Center protocol 102619. All studies utilized C57Bl/6N *Snord116^p−/m+^* mice (B6.Cg-*Snord116^tm1.1Uta^*/J, stock 008149) developed by the Francke lab [29], which were obtained from The Jackson Laboratory and have since been backcrossed onto a C57Bl/6N background over > 10 generations. *Snord116^p−/m+^* study mice (which carry a paternally inherited chromosome 7 lacking the *Snord116* gene cluster) and WT littermates were generated by crossing male C57Bl/6N *Snord116^p−/m+^* mice with female C57BL/6N mice. Housing conditions included a 12 h light–dark cycle and ad libitum water access.

### 2.2. Genotyping

Genotyping was performed using genomic DNA extracted from tail snips and three primers to detect the *Snord*116del and WT *Snord*116 alleles: *Snord*116-M634 (5′-TGGATCTCTCCTTGCTTGTTTTCTC-3′), *Snord*116-M635 (5′-AATCCCCAACCTACTTCAAACAGTC-3′), and *Snord*116-M636 (5′-TTTACGGTACATGACAGCACTCAAG-3′). The following PCR protocol with an annealing temperature at +64 °C generated a WT band of 435 bp and a mutant band of 337 bp [30].

### 2.3. Cell Culture

The HEK293 cells were cultured in high-glucose (4.5 g/L) Dulbecco’s Modified Eagle Medium (DMEM) (Thermo-Fisher Scientific, Waltham, MA, USA) with added L-glutamine, penicillin–streptomycin antibiotic, and fetal bovine serum (FBS). The cells were passaged when they reached 80–90% confluency.

#### 2.3.1. iPSC Lines

The iPSC lines used for this study originated from University of Connecticut Stem Cell Core (UConn Health, Farmington, CT, USA) and are shown in Table 1. Upon arrival, the iPSC lines were adapted to a feeder-free culture following the manufacturer’s instructions (StemCell Technologies, Cambridge, MA, USA) and passaged for an additional 2 passages for the removal of the feeder cells. The feeder-free iPSCs were cultured on Matrigel-coated plates with mTeSR™ Plus medium (StemCell Technologies, Cambridge, MA, USA) and passaged every 3–4 days at a ratio of 1:6–1:10 using Versene (Invitrogen, Waltham, MA, USA). The cells were cultured in a humidified incubator at 37 °C in a 5% CO_2_ atmosphere.

#### 2.3.2. iPSC-Derived Neuronal Induction

iPSCs were induced to become neural progenitor cells (NPCs) according to the monolayer and dual SMAD inhibition protocol [32] using STEMdiff™ SMADi Neural Induction Kit (StemCell Technologies, Cambridge, MA, USA). iPSCs were detached using Accutase to obtain 2 × 10^6^ single cells per well in a 6-well plate pre-coated with Matrigel. Neural induction media were changed daily for 8 days before each passage. To induce the differentiation of NPCs into neural precursor cells (NPreCs), NPCs were detached with Accutase after three passages and plated in 6-well plates coated with 15 µg/mL PLO (Sigma-Aldrich, St. Louis, MO, USA, P3655) and 10 µg/mL Laminin (Sigma-Aldrich, St. Louis, MO, USA, L2020) at a plating density of 105,000 cells per cm^2^. STEMdiff Forebrain Neuron Differentiation medium (STEMCELL Technologies, 08600) was changed daily up to 7 days until confluency reached 80–90%. To generate iPSC-derived neurons, NPreCs were detached with Accutase and plated at 126,000 cells per cm^2^ into STEMdiff Forebrain Neuron Maturation medium (STEMCELL Technologies, 08605). Further maturation into forebrain neurons was conducted by changing the corresponding media every other day until the experiment was carried out.

#### 2.3.3. Mouse Primary Neurons

Mouse primary neurons were isolated from the early postnatal (P0–P2) mouse hippocampus and cortex. The isolation method followed was that described by Beaudoin et al. [33] with minor modification. Briefly, cells from the mouse hippocampus and cortex were dissociated with a solution of trypsin, and the dissociated tissue was added on top of a 4% BSA cushion, followed by centrifugation at 300× *g* for 7 min at room temperature, as described by Moutin et al. [34]. The cell pellet was resuspended in a plating medium, and the cells were plated at 0.5 × 10^6^ cells per well in 12-well precoated plates with 0.5 mg/mL Poly L-Lysine (Sciencell Research, Carlsbad, CA, USA). The plating medium was removed 4 h after plating and replaced with maintenance media. The cells were cultured until DIV4, with half of the maintenance medium changed every other day prior to transfection.

### 2.4. siRNA Design

Several siRNA duplexes (21 nucleotides for sense and antisense) targeting the mouse *Ehmt2* mRNA and human *EHMT2* mRNA were designed using The Genetic Perturbation Platform from Broad Institute. Using the algorithm from the siRNA Sequence Probability-of-Off-Targeting Reduction online tool https://sispotr.icts.uiowa.edu/ (accessed on 1 March 2021), siRNA candidates were checked for Off-Targets in human and mouse RNA sequences, and the best matches were selected with the lowest Probability-of-Off-Targeting-Sites (POTS) values [35].

### 2.5. Plasmid Construct

Five siRNA sequences (Table 2; 21 nucleotides for sense and antisense) with silencing activities against human *EHMT2* and mouse *Ehmt2* coding regions were selected for constructing shRNA silencing plasmids, named U6-shRNA (h*EHMT2*/m*Ehmt2*-11) U6-shRNA (h*EHMT2*/m*Ehmt2*-12), U6-shRNA (h*EHMT2*/m*Ehmt2*-13), U6-shRNA (h*EHMT2*/m*Ehmt2*-14), and U6-shRNA (h*EHMT2*/m*Ehmt2*-15). A negative control without silencing activity against human *EHMT2* and mouse *Ehmt2* coding regions was prepared and named U6-shRNA (Scramble). Each shRNA-expressing construct is driven by a Pol III U6 promoter. Each plasmid contains an additional cassette for EGFP controlled by the ubiquitous synthetic promoter (JeT) and placed upstream of the U6 promoter.

### 2.6. Cell Transfection with siRNA

PWS UPD iPSCs were transfected with 120 pmol of anti-*EHMT2* siRNAs per well of a 6-well plate, using Lipofectamine RNAiMAX, as described [16]. Cells were transfected a second time 48 h following the first transfection and were harvested 72 h after the initial transfection.

### 2.7. Cell Transfection with Plasmid

HEK293 cells were seeded at 175,000 cells per well in 24-well plates and left to grow for 24 h before the transfection. HEK293 cells were transfected (Thermo-Fisher Scientific, Waltham, MA, USA, 161093) with different shRNA plasmid constructs at a total of 500 ng plasmid/well using PEI MAX (Polysciences, Warrington, PA, USA, 24765-1) at a ratio PEI:DNA of 4:1. The PEI:DNA complexes were prepared by mixing the PEI into a diluted solution of DNA with OptiMEM, and the mixture was left to incubate for 10 min before being added to the cells. The medium was replaced 24 h post transfection. Then, 72 h after transfection, cell lysates for RNA were harvested.

The iPSCs were transfected using the reverse transfection method following the manufacturer instructions. The TransIT-LT1 (Mirus Bio, Madison, WI, USA) was used to reverse-transfect 0.25 × 10^6^ iPSCs with the shRNA plasmid constructs. Reverse transfections were performed in 24-well plates using 1.5 µL of TransIT-LT1 to deliver 0.5 µg of DNA (3:1, reagent: DNA) in mTeSR™ Plus with 10 µM rock inhibitor for 4 h, after which the media was replaced with fresh mTeSR™ Plus with 10 µM rock inhibitor. The cells were incubated for 72 h until being harvested.

### 2.8. Transduction of iPSC-Derived Cells with Lentivirus

Non-integrating lentiviral vectors (NILVs) were designed by the VectorBuilder (Chicago, IL, USA) and are U6-based shRNA knockdown vectors containing the EGFP sequence driven by JeT promoter. The NILV-U6-shRNA candidates NILV-U6shRNA (h*EHMT2*/m*Ehmt2*-11) and NILV-U6shRNA (h*EHMT2*/m*Ehmt2*-14) targeting conserved mouse and human *EHMT2* sequences and a control NILV-U6shRNA-Scramble were prepared.

NPreC derived from iPSC UPD and iPSC SD was detached with Accutase, and 240,000 cells per well were plated on 24-well plates coated with PLO and laminin. Mature neurons were generated for 15 days by feeding every other day with STEMdiff Forebrain Neuron Maturation medium (STEMCELL Technologies, 08605). NILVs were added to the wells at a range of viral concentrations [represented as multiplicity of infection (MOI)]. The media was replaced after 24 h of transduction, and the cells were cultured until harvest at the indicated time points. 

### 2.9. Quantitative Real-Time Reverse Transcription (qRT)-PCR

Total RNA extraction from HEK293 cells, iPSCs, and mouse primary neurons was carried out using the RNeasy mini Kit (QIAGEN, Germantown, MA, USA). Briefly, the cells were lysed by adding 350 µL buffer RLT containing mercaptoethanol per well and stored at −80 °C until further extraction was carried out. The samples were then processed according to the manufacturer’s instructions. The RNA concentration was measured by NanoDrop One (Thermo-Fisher Scientific, Waltham, MA, USA). cDNA was prepared using the iScript cDNA Synthesis Kit (BioRad, Hercules, CA, USA) with 1ug of RNA input. Total RNA extraction from iPSC-derived neuron cells was carried out using the miRNeasy Micro kit (QIAGEN, Hilden, Germany).

The qRT primers used for expression analyses are given in Table 3. The EIF4A2 gene was used as the internal control for RNA expression. Expression was quantified by the ΔΔCt method using target-specific and control primers. Data are presented as the relative quantity of targets, normalized with respect to internal control, and relative to the calibrator control sample.

### 2.10. Western Blot Analysis

Total protein from UPD iPSCs was extracted by lysing the cells in cell lysis buffer (RIPA buffer: 25 mM Tris-HCl–pH 7.6, 150 mM NaCl, 1% NP-40, 1% sodium deoxycholate, 0.1% sodium dodecyl sulfate) containing 1X Protease Inhibitor Cocktail (Cell Signaling Technologies, Danvers, MA, USA). The cell lysate was cleared after centrifugation (12,000× *g*) for 10 min at +4 °C. Protein concentrations were determined by using the Pierce bicinchoninic acid protein assay kit (Thermo-Fisher Scientific, Waltham, MA, USA). Equal amounts of total protein (15–25 μg) from cells were subjected to SDS-PAGE. Proteins were then transferred to a polyvinylidene difluoride (PVDF) membrane using the Trans-Blot Turbo Mini 0.2 µm PVDF Transfer Packs (BioRad, Hercules, CA, USA). Membranes were blocked for 1 h at room temperature (RT) in EveryBlot Blocking Buffer (BioRad, Hercules, CA, USA). The membranes were then probed with primary antibody anti-EHMT2 antibody (#3306, Cell Signaling) at dilution 1:500 in EveryBlot Blocking Buffer and incubated overnight at +4 °C. The membranes were then washed with Tris-buffered saline with 0.1% TWEEN 20 (TBS-T) three times and incubated for 1h at RT with goat anti-rabbit StarBright blue 700 secondary antibody (BioRad, Hercules, CA, USA) and hFAB™ Rhodamine Anti-GAPDH antibody (BioRad, Hercules, CA, USA, Cat#: 12004167), and diluted at 1:2500 and at 1:2000 in EveryBlot Blocking Buffer, respectively. The membranes were washed with TBS-T three times, and the immunoreactive bands were visualized by the ChemiDoc™ MP System and analyzed using ImageJ software version 1.54j (National Institute of Health, Bethesda, MD, USA).

#### Statistics

GraphPad Prism 10.2.3 (Boston, MA, USA) was used for statistical analysis. T-test comparisons were used with two pairs of conditions. Unless otherwise indicated in the Figure legends, we analyzed three biological replicates for each data point in all graphs, and the level of significance was as follows: *, *p* < 0.05; **, *p* < 0.01; ***, *p* < 0.001; ****, *p* < 0.0001; ns, *p* > 0.05.

## 3. Results

### 3.1. siRNA Knockdown of EHMT2 in PWS-UPD iPSCs

**Figure 1:**
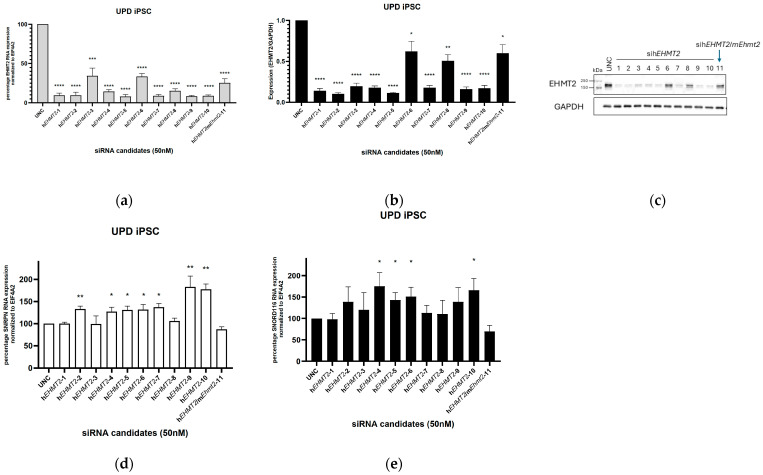
Efficacy of *EHMT2* transcript reduction with specific siRNAs in human UPD patient-derived iPSCs. (**a**). Gene expression of *EHMT2* in PWS UPD iPSCs following transfection with control siRNAs (UNC) or specific siRNAs targeting *EHMT2*. A significant reduction in EHMT2 was observed with all the siRNA candidates relative to the control; (**b**) quantification of the EHMT2 protein in UPD iPSCs treated with siRNA targeting EHMT2 and compared to treated cells with the control siRNA UNC; (**c**) representative image of EHMT2 protein from UPD iPSCs treated with siRNA candidates analyzed by Western blotting; (**d**) gene expression of *SNRPN* and (**e**) gene expression of *SNORD116* in PWS UPD iPSCs following transfection with control siRNAs (UNC) or specific siRNAs targeting *EHMT2*. *EIF4A2* was used as a control. Student’s *t* test: *, *p* < 0.05; **, *p* < 0.01; ***, *p* < 0.001; ****, *p* < 0.0001; data represent means ± S.E.M (N = 3–4).

Several siRNA oligonucleotides were designed to target human *EHMT2* gene expression alone and conserved human and mouse sequences of *EHMT2*/*Ehmt2*. Universal Control (UNC) siRNA was used as a negative control. The expression levels of human *EHMT2* RNA were reduced by at least 90% by six of the siRNA candidates (#1, 2, 5, 7, 9, and 10), as listed in Figure 1a. siRNA candidate #11, targeting both human and mouse sequences for *EHMT2*/*Ehmt2*, showed about 75% reduced mRNA levels of human *EHMT2*.

We confirmed that EHMT2 protein levels in PWS-UPD iPSCs were reduced post treatment with siRNA targeting *EHMT2*/*Ehmt2* compared to UNC siRNA-treated cells. The blots showed an almost complete ablation of protein levels for EHMT2 with the siRNA targeting human or conserved human and mouse sequences of EHMT2/Ehmt2 (Figure 1c).

Transfection with siRNA targeting the human sequence for *EHMT2* resulted in a less than 2-fold increase in *SNORD116* and *SNRPN* expression levels (Figure 1d,e) compared to the control. 

### 3.2. siRNA Knockdown of Ehmt2 in Primary Mouse Neurons

**Figure 2:**
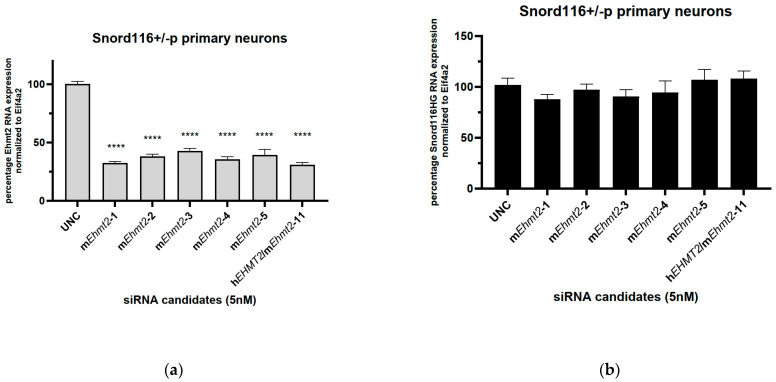
Efficacy of *Ehmt2* transcript reduction with specific siRNAs in mouse primary neurons. (**a**) Gene expression of *Ehmt2* in primary neurons from *Snord116^p−/m+^* mice following transfection with control siRNAs (UNC) or specific siRNAs targeting *Ehmt2*. A significant reduction in Ehmt2 was observed with all the siRNA candidates relative to the control; (**b**) gene expression of *Snord116HG* following transfection with control siRNAs (UNC) or specific siRNAs targeting *Ehmt2*. *Eif4a2* was used as a control. Student’s *t* test: ****, *p* < 0.0001; data represent means ± S.E.M from four biological replicates.

Distinct siRNA oligonucleotides were developed to target the mouse *Ehmt2* sequence only and conserved sequences between the human and mouse *EHMT2*/*Ehmt2* sequences. They are tested on mouse primary neurons from *Snord116^p−/m+^* mice. Universal Negative Control (UNC) siRNA was used as a negative control. The siRNAs targeting the mouse *Ehmt2* sequence reduced the expression levels of mouse *Ehmt2* RNA by nearly 70% at 48 h post transfection (Figure 2a). The siRNA targeting both the human and mouse sequence for *EHMT2*/*Ehmt2* showed an efficiency in inhibiting the expression levels of mouse *Ehmt2* RNA similar to that of the siRNA targeting the mouse sequence only.

Probes to detect transcripts for *Snord116* (Snord116 HG) were used. All siRNA candidates targeting mouse *Ehmt2* including the siRNA for both the mouse and human sequence showed no significant increase in *Snord116 HG* RNA expression levels (Figure 2b) in the primary neurons of mouse *Snord116^p−/m+^*. 

### 3.3. shRNA Knockdown of EHMT2 in HEK293 Cells

**Figure 3:**
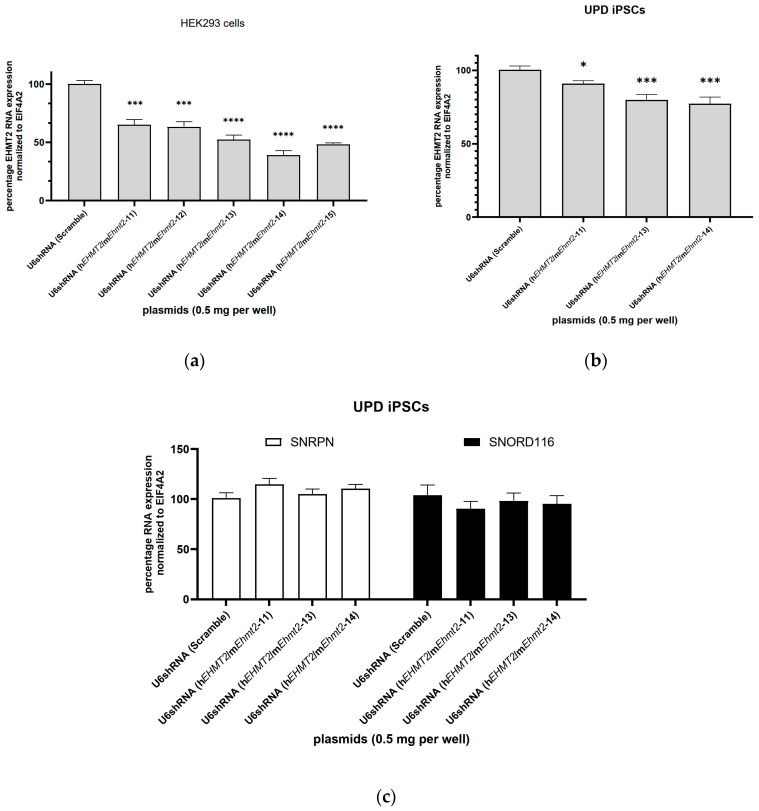
Expression levels of *EHMT2*, *SNRPN*, and *SNORD116* after transfection with U6 anti-*EHMT2* shRNA DNA plasmids: (**a**) HEK293 cells were transfected with a plasmid construct control (shRNA Scramble) and shRNA specifically targeting *EHMT2*. A significant reduction in EHMT2 expression was observed with all the shRNA plasmid candidates relative to the control; (**b**) PWS UPD iPSC was transfected with the plasmid control and shRNA targeting *EHMT2*. A moderate but significant reduction in EHMT2 expression was observed 72 h post transfection; (**c**) *SNRPN* and *SNORD116* expression levels in PWS UPD iPSC remained at the same level as for the control after transfection with U6shRNA plasmids targeting *EHTM2*. Student’s *t* test: *, *p* < 0.05; ***, *p* < 0.001; ****, *p* < 0.0001; data represent the means ± S.E.M from three biological replicates.

We designed four DNA plasmids with shRNA sequences targeting both human and mouse *EHMT2* (named U6-shRNA(h*EHMT2*/m*Ehmt2*-12), U6-shRNA(h*EHMT2*/m*Ehmt2*-13), U6-shRNA(h*EHMT2*/m*Ehmt2*-14), and U6-shRNA(h*EHMT2*/m*Ehmt2*-15). To ensure the functionality of our plasmid constructs, we transfected into HEK293 cells the plasmid constructs encoding for shRNA targeting conserved mouse and human *EHMT2* sequences and driven under the U6 promoter. The plasmid construct encoding for the shRNA sequence named scramble is used as a control. All the shRNA candidates significantly reduced the RNA levels of EHMT2 in HEK293 cells 72 h post transfection from 35% up to 60% (Figure 3a).

### 3.4. shRNA Knockdown of EHMT2 in PWS-iPSCs

The same shRNA plasmid constructs—shRNA(h*EHMT2*/m*Ehmt2*-13) and U6-shRNA(h*EHMT2*/m*Ehmt2*-14)—that were tested in HEK 293 cells were tested in PWS UPD iPSCs (Figure 3b,c). An additional plasmid construct containing sihEHMT2/mEhmt2-11 was added for comparison. The RNA levels for *SNORD116* and *SNRPN* were measured 72 h post transfection. No statistically significant change in *SNORD116* and *SNRPN* expression levels were measured compared to the control with shRNA Scramble (Figure 3c), despite a ~23% reduction in *EHMT2* transcript levels with the U6-shRNA(h*EHMT2*/m*Ehmt2*-14) plasmid construct (Figure 3b).

### 3.5. shRNA Knockdown of EHMT2 in PWS-iPSC-Derived Neurons 7 Days and 14 Days Post Transduction with Lentiviral Constructs

Non-integrative lentiviral constructs were designed encoding for the shRNA(h*EHMT2*/m*Ehmt2*-11) and shRNA(h*EHMT2*/m*Ehmt2*-14) targeting mouse *Ehmt2* and human *EHMT2* and were applied to mature iPSC-derived neurons from two different PWS patient cell lines (UPD and SD).

**Figure 4:**
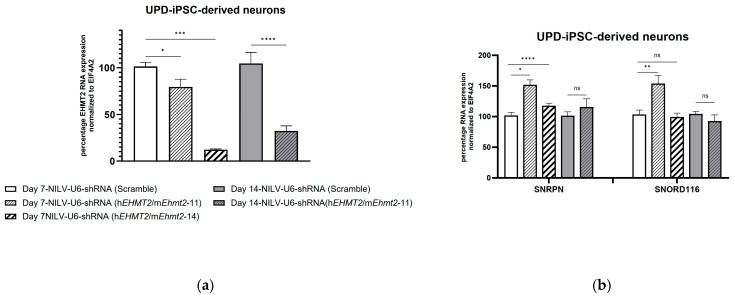
Expression levels of *EHMT2*, *SNRPN* and *SNORD116* in UPD iPSC-derived neurons transduced with lentivirus containing anti-*EHMT2* shRNA or control (Scramble): (**a**) NILV-U6shRNA(h*EHMT2*/m*Ehmt2*-14) significantly reduced *EHMT2* RNA levels as opposed to a mild reduction with NILV-U6shRNA (h*EHMT2*/m*Ehmt2*-11) 7 days post transduction; *EHMT2* expression is further reduced 14 days post transduction with NILV-U6shRNA (h*EHMT2*/m*Ehmt2*-11). (**b**) NILV-U6shRNA (h*EHMT2*/m*Ehmt2*-11) significantly increased *SNRPN* and *SNORD116* transcript levels 7 days post transduction, as opposed to no change 14 days post transduction. There is a significant increase in SNRPN transcripts levels without any change in *SNORD116* transcripts levels 7 days post transduction with NILV-U6shRNA (h*EHMT2*/m*Ehmt2*-14). Student’s *t* test: *, *p* < 0.05; **, *p* < 0.01; ***, *p* < 0.001; ****, *p* < 0.0001; ns, *p* > 0.05; data represent the means ± S.E.M of four biological replicates.

Seven days post transduction with NILV constructs, *EHMT2* RNA levels were significantly downregulated in UPD-PWS iPSC neurons from 14% to 88% with NILV-U6- shRNA(h*EHMT2*/m*Ehmt2*-11) and NILV-U6- shRNA(h*EHMT2*/m*Ehmt2*-14), respectively (Figure 4a).

The levels of *SNRPN* and *SNORD116* significantly increased by about 50% in the PWS UPD iPSC-derived neurons seven days post transduction with NILV-U6 shRNA(h*EHMT2*/m*Ehmt2*-11) compared to the control (Figure 4b). A slight increase in RNA levels for *SNRPN* (about 17%) was observed in PWS UPD iPSC-derived neurons treated with NILV-U6 shRNA(h*EHMT2*/m*Ehmt2*-14), whereas no increase in *SNORD116* RNA expression level was measured (Figure 4b).

Fourteen days post transduction with NILV-U6, shRNA(h*EHMT2*/m*Ehmt2*-14) induced toxicity in UPD-iPSC-derived neurons, and no data were collected for this time point. However, we were able to collect samples 14 days post transduction from UPD iPSC-derived neurons transduced with NILV-U6 shRNA(h*EHMT2*/m*Ehmt2*-11). The results showed about 70% downregulation of *EHMT2* RNA levels (Figure 4b), with no concomitant change in RNA levels for *SNRPN* and *SNORD116* (Figure 4b).

**Figure 5:**
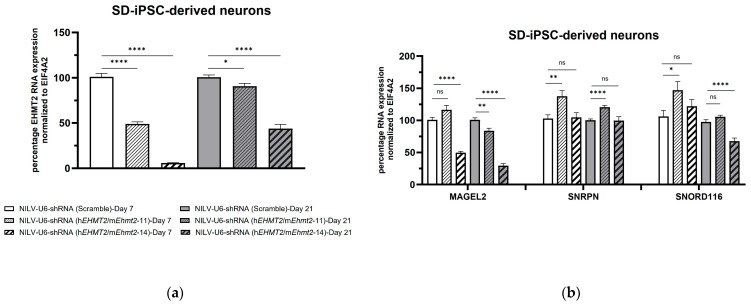
Expression levels of *EHMT2, MAGEL2, SNRPN*, and *SNORD116* in SD-iPSC-derived neurons transduced with lentivirus with shRNA targeting *EHMT2* and control (Scramble): (**a**) NILV-U6shRNA(h*EHMT2*/m*Ehmt2*-14) and NILV-U6shRNA (h*EHMT2*/m*Ehmt2*-11) significantly reduced *EHMT2* RNA levels 7 days post transduction as opposed to a milder reduction 21 days post transduction; (**b**) NILV-U6shRNA (h*EHMT2*/m*Ehmt2*-11) significantly increased *SNRPN* transcript levels 7 days and 21 days post transduction as opposed to no change in *SNRPN* transcript levels 7 days and 21 days post transduction with NILV-U6shRNA(h*EHMT2*/m*Ehmt2*-14). A significant increase in SNORD116 transcripts levels is shown at day 7 post transduction with NILV-U6shRNA (h*EHMT2*/m*Ehmt2*-11). Student’s *t* test; *, *p* < 0.05; **, *p* < 0.01; ****, *p* < 0.0001; ns, *p* > 0.05; data represent the means ± S.E.M from four biological replicates.

In PWS SD-iPSC-derived neurons, *EHMT2* RNA levels were significantly reduced seven days post transduction from 50% to 96% with NILV-U6 shRNA(h*EHMT2*/m*Ehmt2*-11) and NILV-U6 shRNA(h*EHMT2*/m*Ehmt2*-14), respectively (Figure 5a). To our surprise, we observed a significant increase in SNRPN and *SNORD116* RNA levels from 37% to 47%, respectively (Figure 5b), compared to the control with NILV-U6 shRNA(h*EHMT2*/m*Ehmt2*-11) in PWS SD-iPSC-derived neurons seven days post transduction. However, no significant increase in RNA levels for *SNRPN* and *SNORD116* was measured with NILV-U6 shRNA(h*EHMT2*/m*Ehmt2*-14), despite the highest decrease in EHMT2 RNA expression levels being observed.

The expression levels of *MAGEL2* were significantly reduced by ~50% with NILV-U6 shRNA(h*EHMT2*/m*Ehmt2*-14), whereas no significant changes were observed in *MAGEL2* RNA levels in SD-iPSC-derived neurons seven days post transduction with NILV-U6 shRNA(h*EHMT2*/m*Ehmt2*-11) (Figure 5b).

### 3.6. shRNA Knockdown of EHMT2 in PWS 2-9-iPSC-Derived Neurons 21 Days Post Transduction with Lentiviral Constructs

Twenty-one days post transduction with shRNA lentiviral constructs, *EHMT2* RNA levels were significantly downregulated by about 10% to 60% with NILV-U6 shRNA(h*EHMT2*/m*Ehmt2*-11) and NILV-U6 shRNA (h*EHMT2*/m*Ehmt2*-14), respectively (Figure 5a). The levels of expression for *SNRPN* significantly increased by 20% with NILV-U6 shRNA(h*EHMT2*/m*Ehmt2*-11) compared to the control, whereas no changes were observed with NILV-U6 shRNA (h*EHMT2*/m*Ehmt2*-14) (Figure 5b). No significant differences in *SNORD116* RNA levels were observed with NILV-U6 shRNA(h*EHMT2*/m*Ehmt2*-11) compared to the control whereas about a 30% significant decrease in SNORD116 RNA levels was observed with NILV-U6 shRNA (h*EHMT2*/m*Ehmt2*-14) compared to the control (Figure 5b). Twenty-one days post transduction with shRNA lentiviral constructs, the expression levels for *MAGEL2* were significantly reduced to 20% and to 70% with NILV-U6 shRNA (h*EHMT2*/m*Ehmt2*-11) and NILV-U6 shRNA (h*EHMT2*/m*Ehmt2*-14), respectively (Figure 5b).

## 4. Discussion

We measured PWS gene transcripts such as *Snord116*/*SNORD116, SNRPN*, and *MAGEL2* in PWS patient-derived iPSCs, PWS patient iPSC-derived neurons, and mouse primary neurons to assess maternal activation with anti-*EHMT2* RNAi and therapeutic feasibility. We demonstrated that several siRNA oligonucleotides targeting *Ehtm2*/*EHMT2* significantly decreased the transcript and protein expression levels of Ehmt2/EHMT2 in both mouse *Snord116* deletion primary cortical neurons and human PWS iPSC lines. Several plasmid constructs encoding for shRNA, which targets *EHMT2*, also efficiently inhibited the expression levels of the EHMT2 protein in HEK293 cells. The lentiviral delivery of anti-*EHMT2* shRNA produced a sustained reduction in *Ehmt2*/*EHMT2* in paternally deleted *Snord116* mouse primary neurons and PWS patient-derived iPSCs (up to 90% downregulation of *ehmt2*/*EHMT2* transcript levels). In *Snord116*-deletion mice, we observed no increase in *Snord116* transcript levels with *Ehmt2* RNA interference. In UPD patient iPSC-derived neurons, time-dependent increases in SNRPN and *SNORD116* transcript levels were observed; in SD patient iPSC-derived neurons, a statistically significant increase in *SNRPN* and a time-dependent increase in *SNORD116* were observed; and *MAGEL2* exhibited a sustained decrease in transcript levels. Taken together, our results show that inhibiting the expression of EHMT2 could partially activate the maternal expression of *SNRPN* and *SNORD116* in PWS iPSCs.

Several studies have described the manipulation of the epigenetic regulation of PWS genes. Zinc finger protein ZNF274 inhibition induced histone modification in PWS patient iPSC-derived neurons and induced about a 20-fold increase in *SNORD116* transcription levels relative to controls [36]. However, the increase was still approximately 1000-fold lower compared to PWS iPSC from healthy patients. Kim et al. showed that by inhibiting EHMT2 activity with small inhibitors such as UNC0638, activation of maternal expression PWS genes *SNORD116* and *SNRPN* in fibroblasts from PWS patients (5–6 Mb deletion of the paternal copy of the 15q11-q13 region) approached healthy level controls [24]. Our approach was similar in targeting *Ehmt2*/*EHMT2* but with a translationally relevant sustainable reduction.

In primary neurons from mice with *Snord116* paternal deletion, the expression of downstream noncoding exons of *Snurf*/*Snrpn* are specifically expressed in neuronal cells [37]. We did not observe an induction of maternal *Snord116* host gene expression levels in mouse primary neuronal cells from *Snord116*-deletion mice despite an siRNA-induced 70% inhibition of *Ehmt2* mRNA expression. Kim et al. [24] have, however, identified small inhibitors of Ehmt2 capable of activating the maternal expression of *Snrpn-EGFP* in mouse embryonic fibroblasts carrying the *Snrpn-EGFP* fusion gene on the maternal chromosome. This might be explained by the fact that post-transcriptional modification resulting from reduced levels of Ehmt2 was not seen in our experimental procedure and cell type due to the limited exposure time of the anti-*Ehmt2* siRNA.

To study a longer exposure in reducing *EHMT2* in human neurons and its effects, we utilized an anti-*EHMT2* shRNA as a lentiviral construct. We found a maternal activation of PWS maternal alleles for *SNRPN* and *SNORD116* but no changes in *MAGEL2* mRNA levels after one week of treatment with NILV EHMT2 in PWS UPD iPSC-derived neurons. The regulation of *MAGEL2* expression is described as being regulated by the PWS-IC according to a mouse study investigating a paternally inherited deletion of the chromosome 7 PWS imprinting center [38]. However, the maternal expression of *Magel2* was not described in the neurons of PWS mice treated with small inhibitors of EHMT2 [24]. Langouet et al. [36] described the maternal activation of *MAGEL2* from PWS UPD patients from specifically knocking down ZNF274 [36,39]. Based on our work, the shRNA knockdown of *EHMT2* is not enough to activate the maternal expression of *MAGEL2* in UPD patient iPSC-derived neurons. A stable knockdown of zinc finger ZNF274 for 10 weeks in UPD iPSC-derived neurons restored *SNORD116* and *MAGEL2* to healthy levels [36,40]. Therefore, the data suggest that ZNF274, not EHMT2, is a regulatory element for *MAGEL2*.

The RNAi of *EHMT2* resulted in the activation of maternally silenced *SNRPN* and *SNORD116* transcripts in specific lines of PWS patient iPSC-derived neurons. We were unsuccessful in maintaining PWS UPD mature neurons beyond one week of treatment with anti-*EHMT2* shRNA lentiviral exposure. We were able to maintain mature PWS SD neurons in culture for 3 weeks of treatment following anti-*EHMT2* shRNA lentiviral exposure. Although the maternal activation of *SNRPN* was observed after 3 weeks of culture, a similar effect was not observed for *SNORD116*. This could be explained by the fact that the inhibitory effect of anti-*EHMT2* shRNA via lentivirus was less pronounced 3 weeks later and that other mechanisms such as ZNF274 act independently of the PWS-IC in maintaining *SNORD116* silencing on the maternal allele [16,36]. Paradoxically, 3 weeks after the incubation of anti-*EHMT2* lentivirus in PWS SD mature neurons, we found that *MAGEL2* transcript levels decreased in PWS SD iPSC-derived neurons. One potential explanation may be a reliance on mechanisms independent of *SNORD116* regulation.

As mentioned above, EHMT2 does not act alone in regulating H3K9 methylation in neurons. Other methyltransferases including SETDB1 and SUV39H1 are shown to modulate the dimethylation of H3K9 [41,42,43]. Moreover, a paralog of EHMT2 called EHMT1 is also important for similar biological phenomena ascribed to EHMT2, such as modulating H3K9 methylation [44,45]. A recent publication described the role of both EHMT2 and EHMT1 knockdown as necessary to efficiently reduce H3K9me2 and exacerbate TNFα-induced lipolysis in mature adipocytes [46]. We recommend that future studies explore this hypothesis by knocking down several methyltransferases, such as SETDB1 and EHMT1.

Currently, the levels of PWS gene expression needed to exert a therapeutic effect on PWS patients are unknown. Unpublished data from our laboratory and others demonstrated approximately a 5000-fold increase in PWS genes in healthy versus PWS patient iPSC-derived neurons. Furthermore, exogenous alterations in the epigenetic regulation of genes in post-mitotic cells are slow compared to similar interrogations in pre-mitotic cells. As such, care must be taken in representing the data as evidence of a translationally relevant therapy. An additional consideration in the transition to human therapy with RNAi is the probability of off-target effects. As described, the siRNAs/shRNAs developed herein were designed with computer algorithms to estimate the probability of off-target binding. However, a detained transcriptomic analysis would be a requisite for safety testing. Further considerations include the burden of treatment through costs, the frequency of treatment, and the translational relevance of the route of administration.

Despite these considerations, recent clinical trials of epigenetic, “stop-the-stop” therapies with antisense oligonucleotides for the treatment of Angelman Syndrome (NCT04259281, NCT05127226), a neurodevelopmental disorder with genetic underpinnings on the same loci as PWS genes, can be considered as a positive advancement towards a similar therapy for PWS. While we would argue that the data presented here argue against singular RNAi targeting of *EHMT2* as therapeutically relevant for PWS, our data confirm a partial role of EHMT2 in the maternal regulation of PWS genes. Furthermore, a combinatorial approach targeting multiple epigenetic regulators through translationally relevant approaches, while reducing the burden on the patient to a single dose, remains a viable consideration for future therapeutic studies.

## 5. Conclusions

Our study showed that reducing EHMT2 expression with either siRNA or shRNA resulted in modest increases in some PWS patient cell lines. However, these increases do not approach healthy control levels, which limits the potential therapeutic effect on PWS of such an approach.

## 6. Patents

A provisional patent has been filed for the anti-*EHMT2* siRNAs/shRNAs and their use (with RKB, HRK, and SJG as co-inventors).

## Figures and Tables

**Table 1:** Prader-Willi Syndrome iPSC lines.

iPSC Lines	Description	Reference	Abbreviation
PWS UPD 1–2	PWS uniparental disomy	[31]	UPD iPSC
PWS 2–9	PWS small deletion	[31]	SD iPSC

**Table 2:** List of shRNA sequences used in this study. hsa, Homo sapiens; mmu, Mus musculus.

shRNA Name	Sequence (5′-3′)—Sense	Sequence (5′-3′)—Antisense	Species Target *EHMT2*
h*EHMT2*/m*Ehmt2*-11	TAAATGTTGGGTTTGGTAATA	TATTACCAAACCCAACATTTA	hsa and mmu
h*EHMT2*/m*Ehmt2*-12	GGCGCAAGGCCAAGAAGAAAT	ATTTCTTCTTGGCCTTGCGCC	hsa and mmu
h*EHMT2*/m*Ehmt2*-13	GCGCAAGGCCAAGAAGAAATG	CATTTCTTCTTGGCCTTGCGC	hsa and mmu
h*EHMT2*/m*Ehmt2*-14	TGATGTGAGAGAGGATGATTC	GAATCATCCTCTCTCACATCA	hsa and mmu
h*EHMT2*/m*Ehmt2*-15	GTGAGAGAGGATGATTCTTAC	GTAAGAATCATCCTCTCTCAC	hsa and mmu

**Table 3:** hsa, Homo sapiens; mmu, Mus musculus; R, reverse; F, forward.

Primer Name	Sequence	Species	SybR or with Probe
EIF4A2 F	CAACGTGCATTGTGCTTCTT	hsa	SybR
EIF4A2 R	ACGACTAACGTCGCTTTGCT	hsa	SybR
SNORD116 F	GGATCGATGATGAGTCCCC	hsa	SybR
SNORD116 R	TCCGATGAGAACGACGGTAT	hsa	SybR
SNRPN F	TTGCTGCGACTGCCAGTATT	hsa	SybR
SNRPN R	GCCCATGGGTGGTCTCATAC	hsa	SybR
MAGEL2 F	ATCTGGAAGCCCAAGAGGAC	hsa	SybR
MAGEL2 R	ACCTGGATAGGGCTTTGGAC	hsa	SybR
EHMT2 F	ATGCAGTGGACAAACAGCAG	hsa	SybR
EHMT2 R	ACCGTCCTCCTCCTTGCTAT	hsa	SybR
Snord116 HG F	TGTTGCTGACTTGCCCTAG	mmu	probe
Snord116 HG R	GTTCGATGGAGACTCAGTTGG	mmu	probe
mSnord116 (probe)	AAACATGCAGAGGAAATGGCCCC	mmu	probe
Eif4	Mm01730183_gH/4448484	mmu	probe
Ehmt2 F	TGCGTACTCTGTGGATGAGC	mmu	SybR
Ehmt2 R	TCTGACTGATTGCCCGACTC	mmu	SybR

## Data Availability

The data presented in this study are available on request from the corresponding author due to an ongoing patent application.
